# Quantifying cost savings from outpatient parenteral antimicrobial therapy programme: a systematic review and meta-analysis

**DOI:** 10.1093/jacamr/dlaf049

**Published:** 2025-04-08

**Authors:** Solomon Ahmed Mohammed, Jason A Roberts, Manuel Mirón-Rubio, Luis Eduardo López Cortés, Getnet Mengistu Assefa, James Pollard, Kate McCarthy, Mark Gilchrist, Menino Cotta, Fekade B Sime

**Affiliations:** UQ Centre for Clinical Research, The University of Queensland, Brisbane, QLD, Australia; Department of Pharmacy, Wollo University, Dessie, Ethiopia; UQ Centre for Clinical Research, The University of Queensland, Brisbane, QLD, Australia; Department of Pharmacy and Intensive Care Medicine, Royal Brisbane and Women’s Hospital, Brisbane, QLD, Australia; Herston Infectious Disease Institute (HeIDI), Metro North Health, Brisbane, QLD, Australia; Division of Anaesthesiology Critical Care Emerging and Pain Medicine, Nimes University Hospital, University of Montpellier, Nîmes, France; Hospital Universitario de Torrejón, Unidad de Hospitalización a Domicilio, Madrid, Spain; Unidad Clínica de Enfermedades Infecciosas y Microbiología, Instituto de Biomedicina de Sevilla (IBiS)/CSIC, Seville, Spain; Hospital Universitario Virgen Macarena, and Departamento de Medicina, Universidad de Sevilla, Seville, Spain; CIBERINFEC, Instituto de Salud Carlos III, Madrid, Spain; UQ Centre for Clinical Research, The University of Queensland, Brisbane, QLD, Australia; Department of Pharmacy, Wollo University, Dessie, Ethiopia; Cabrini @ Home, Cabrini Health, Melbourne, Australia; Royal Brisbane Clinical School, Faculty of Medicine, The University of Queensland, Brisbane, QLD, Australia; Department of Infectious Diseases, Royal Brisbane and Women’s Hospital, Brisbane, QLD, Australia; Department of Pharmacy/Infection, Imperial College Healthcare NHS Trust, London, UK; Department of Infectious Diseases, Imperial College, London, UK; UQ Centre for Clinical Research, The University of Queensland, Brisbane, QLD, Australia; UQ Centre for Clinical Research, The University of Queensland, Brisbane, QLD, Australia

## Abstract

**Background:**

The outpatient parenteral antimicrobial therapy (OPAT) programme was introduced to reduce costs and enhance the quality of life for patients requiring prolonged treatment with parenteral antimicrobials. However, given the escalating inflation, the extent of current cost savings achieved through OPAT programmes remains unclear. This systematic review and meta-analysis employ a cost-minimization analysis to quantify the cost savings from OPAT compared to inpatient treatment.

**Methods:**

The Cochrane Library, MEDLINE, Embase, PubMed and Web of Science databases were searched for studies comparing the costs of parenteral antimicrobial treatment without restriction on study design and year. Two reviewers conducted eligibility screening and cross-validated the extracted data. The cost data were adjusted and inflated to 2023 US dollars. A random effect model calculated mean differences (MD) with 95% confidence intervals (CI). The review protocol was registered on PROSPERO (CRD42024584201).

**Results:**

Twenty studies involving 2790 patients were included in the systematic review, and six studies (three randomized controlled trials and three cohorts) were subject to metanalysis. Collectively, these included 560 patients who received treatment in outpatient settings, and 491 treated as inpatients. The cost of parenteral antimicrobial per episode of care was lower in the outpatient settings MD −$5436.73 (95% CI: −$9589.24 to −$1284.22, I² = 96%; *P* = 0.01) than in inpatient settings.

**Conclusions:**

OPAT significantly saves costs compared to inpatient treatment. We recommend comprehensive analysis of treatment costs from all perspectives, including various cost types.

## Introduction

Infections are a major public health challenge worldwide.^1^ Traditionally, serious infections have been treated with parenteral antimicrobials administered in a hospital environment. However, there has been a broad rise in infections leading to increased hospitalization rates, straining healthcare facilities, and elevating the risk of nosocomial infections.^2^ Avoiding hospitalization or facilitating early discharge requires the implementation of an effective stewardship programme.^3^

Outpatient parenteral antimicrobial therapy (OPAT) is a care delivery modality that avoids hospitalization.^4,5^ Since its implementation in 1974,^6^ the programme has been offered in various ambulatory settings, including infusion centres, hospital-based clinics, skilled nursing facilities and patient’s homes.^7^ It is indicated for patients in clinically stable conditions who have the necessary support.^8^ The disease conditions treated in the programme include skin and soft tissue infections, bone and joint infections, bacteraemia, wound infections, urinary tract infections, intra-abdominal infections and central nervous system infections.^9^

Treating these conditions in outpatient settings is both safe and effective compared to traditional inpatient treatments.^10^ OPAT is associated with high patient satisfaction, as it allows patients to receive treatment in the comfort of their homes, engage with family during treatment and maintain their daily routines.^11^ Additionally, OPAT reduces costs by freeing up hospital beds, thereby improving patient flow and increasing the capacity for new admissions.^12^ It also minimizes the income and productivity loss associated with prolonged hospitalization by facilitating an earlier return to work.^13–17^ Even though the savings from the OPAT programme vary depending on the type of patients, the healthcare system, payers and society,^18^ there is potential for further cost reductions through the advancements of OPAT models of care.^19^ However, there is a lack of a clear description regarding the additional cost savings of the OPAT programme. Consequently, the extent of cost savings from the OPAT programme compared to inpatient therapy remains uncertain.^3^

Psaltikidis *et al*. conducted a review in 2017 to evaluate the cost savings of the OPAT programme. However, the studies included in their review were heterogeneous, and the currency was neither adjusted for inflation nor standardized to a common currency, which precluded a robust meta-analysis.^20^ Thus, there is a lack of reviews that have pooled cost data comparing the savings associated with OPAT to those from inpatient treatment. Such economic evaluations are crucial for delivering optimal care while ensuring financial viability within the healthcare system. Therefore, this systematic review and meta-analysis assessed the cost savings of OPAT compared to inpatient treatment by inflating and adjusting to a common currency.

## Methods

### Search strategy

A systematic search was conducted across the Cochrane Library, MEDLINE, Embase, PubMed and Web of Science databases. A manual search of the reference lists of included studies and review articles was also conducted to identify further relevant literature. The complete search strategy is detailed in Table S1 (available as Supplementary data at *JAC-AMR* Online). No restrictions were imposed on language, publication year, or format. This review adhered to the Preferred Reporting Items for Systematic Reviews and Meta-Analyses (PRISMA) guidelines,^21^ and the protocol was registered on PROSPERO (CRD42024584201).

### Study selection

Studies were included if they compared the cost, cost–effectiveness, cost–benefit, or cost–utility of inpatient parenteral antimicrobial therapy with OPAT. Studies on OPAT included both early discharge facilitation and admission avoidance. The study population included all patients receiving parenteral antimicrobials in outpatient or inpatient settings. Patients receiving parenteral antimicrobials in the outpatient setting began their treatment either while admitted to the hospital or at the emergency department, and subsequently completed their treatment in an ambulatory setting, such as fixed clinics, emergency departments, or their own homes. Inpatient patients were those who completed their treatment while remaining in hospital beds.

Studies that relied on assumption-based imputation and those that did not distinguish between oral and parenteral antimicrobial treatment were excluded. Additionally, non-peer-reviewed studies, abstracts and reviews were excluded.

### Screening of studies

Two independent reviewers (S.A.M. and G.M.A.) screened the abstracts and full-text studies based on predefined criteria. The screening process was facilitated by Covidence (Veritas Health Innovation, Melbourne, Australia). Any conflicts between the reviewers were resolved through discussion, involving a third reviewer, F.S., when necessary. The Kappa statistic was used to assess and report the agreement between the two reviewers.

### Data extraction

Data were extracted using a standardized form, which included study characteristics (year, country, design, authors, currency type, type of economic evaluation, disease condition and antimicrobials), participant characteristics (number, age, gender) and study outcomes (cost, days of hospitalization avoided and health outcomes). The studies defined health outcomes differently, and these were extracted as reported. One reviewer (S.A.M.) performed the data extraction, while the senior reviewer (F.S.) verified the extracted data.

### Risk of bias assessment

One reviewer (S.A.M.) assessed the risk of bias of non-randomized studies using Newcastle–Ottawa Scale,^22^ while the Cochrane risk of bias assessment tool was used for randomized studies.^23^

### Data analysis

A cost-minimization analysis was conducted due to the similar clinical outcomes between OPAT and inpatient treatment.^10^ The results of the review were summarized as follows: (i) the percentage of cost savings per patient for OPAT was calculated by subtracting the average OPAT cost from the average hospitalization cost and dividing by the average hospitalization cost; (ii) the overall average cost savings percentage was determined by dividing the total OPAT cost savings from all studies by the number of studies; and (iii) the average number of hospitalization days avoided was calculated by dividing the total number of hospitalization days averted by the total number of patients or treatment episodes. Detailed calculations and the extracted cost data are presented in Table S2.

The cost data reported across different years and currencies were standardized to a target currency (USD) and year (2023) through a two-stage process.^24^ In the first stage, the original cost data are adjusted from its initial price year to the target price year using the International Monetary Fund (IMF) Gross Domestic Product (GDP) Deflator Index to account for inflation. For studies that did not specify a price year, the last year of patient recruitment was used as the reference price year. In the second stage, the price–year-adjusted cost data are converted from the original currency to the target currency using the Purchasing Power Parities (PPP) for GDP from the IMF World Economic Outlook Database. The PPP adjusts for differences in price levels between countries.^24^ This cost standardization was performed using a web-based tool, CCEMG–EPPI Centre Cost Converter (v.1.7).

Studies that reported the mean and standard deviation (SD) of cost data were included in the meta-analysis. The SD was calculated for studies that provided the standard error of the mean. The data were extracted as presented and incorporated into the narrative for studies reporting median and range. The mean cost difference for parenteral antimicrobial treatment was calculated using the Meta package in R software (version 4.3.0) (Posit Software, Vienna, Austria). A random effects model (Mantel–Haenszel method) was employed for the analyses.^25^ The I-squared statistic was used to assess heterogeneity among included studies, with a value greater than 50% indicating substantial heterogeneity.^26^ Subgroup analyses were conducted to evaluate variations in OPAT care models: S-OPAT (self or carer-administered OPAT at patients’ homes), H-OPAT (physician or nurse-administered OPAT at patients’ homes) and C-OPAT (outpatient clinic or infusion centre administration). A sensitivity analysis was performed to test the robustness of the primary analysis by restricting it to studies that evaluated similar types of costs (direct and indirect), cost perspectives (health care provider, payer and society), population (adult and paediatric), ways of hospitalization avoidance (admission avoidance and facilitation of discharge) and those published after 2000. Publication bias could not be assessed because a funnel plot requires at least 10 studies.^26^

## Results

### Search results

**Figure 1.**
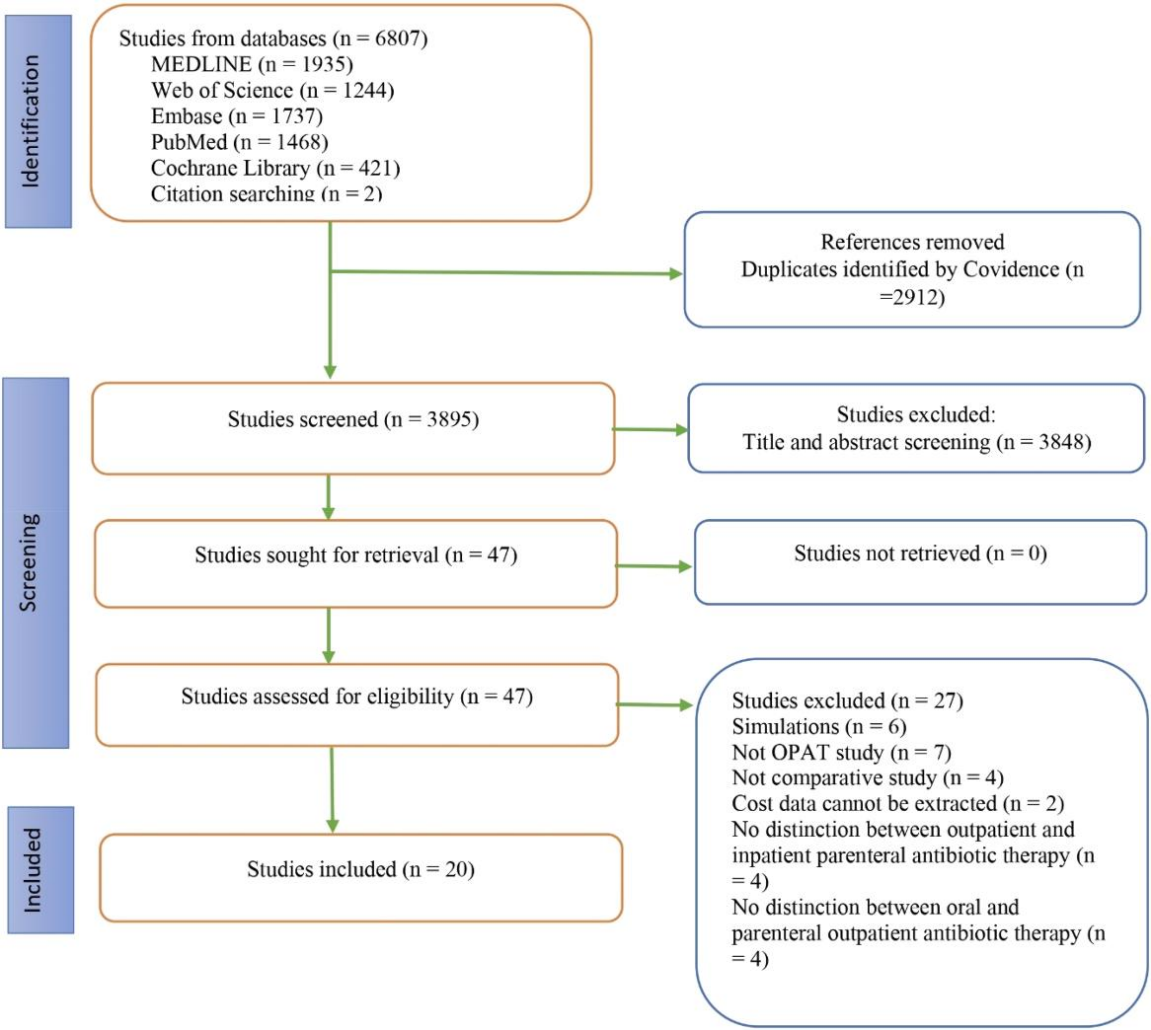
Study eligibility screening.

The screening process for the studies is presented in Figure 1. A total of 6807 studies were identified from all databases. After removing 2912 duplicate studies, 3895 were screened for title and abstract. The full length of 47 studies was assessed, and 20 were included in the final analysis.^27–46^ The level of agreement between the two reviewers was 70.25% for title and abstract screening and 95.98% for full-length screening. The list of excluded studies and the reasons for their exclusion is presented in Table S3.

### Characteristics of included studies

The characteristics of the included studies are presented in Table 1. These studies were conducted between 1978^28^ and 2022,^44^ with two-thirds taking place in Australia^32–35,42,43^ and the USA.^28–31,39–41^ Among the studies, five were randomized controlled trials (RCTs),^27,31,34,42,43^ eight were prospective cohorts^28–30,33,37,39,40,46^ and seven were retrospective cohorts,^32,35,36,38,41,44,45^ involving a total of 2790 participants. The average percentage of male participants was 51.87% in the OPAT group and 47.49% in the inpatient group. The mean age of participants was 31.88 years in the OPAT group and 32.08 years in the inpatient group.

**Table 1.** Characteristics of included articles

Author and year	Country	Study design	Quality^a^	Number of participants		Male %		Mean age	
				OPAT	IPAT	OPAT	IPAT	OPAT	IPAT
Hendricks 2011^31^	USA	RCT	Some concern	35	57	54	29	47.1	46.1
Hensey 2017^32^	Australia	Retrospective cohort	8	41	130	61	30.8	NR	NR
Martel 1994^37^	Canada	Prospective cohort	8	33	17	NR	NR	NR	NR
Antoniskis 1978^28^	USA	Prospective cohort	9	13	7	61.5	57.1	37.2	52.7
Kameshwar 2016^35^	Australia	Retrospective cohort	8	124	204	64.5	54.9	59^b^	58.5^b^
Donati 1987^29^	USA	Prospective cohort	9	26	38	46.1	47.4	23.3	23.3
Warner 1998^41^	USA	Retrospective cohort	8	120	122	67.6	55.14	11.2	11.5
Yong 2008^45^	Singapore	Retrospective cohort	9	69	93	52.8	50.5	53	56
Ramasubramanian 2018^38^	India	Retrospective cohort	8	90	10	43		56.2	
Wolter 2004^43^	Australia	RCT	High	44	38	45.5	34.2	43^b^	49^b^
Ahmed 2007^27^	Egypt	RCT	High	61	58	59	60.3	7.2	6.3
Talcott 1994^40^	USA	Prospective cohort	7	30	27	43.3	NR	38^b^	NR
Fishman 2000^30^	USA	Prospective cohort	8	52	98	NR	NR	10.2	
Lacroix 2014^36^	France	Retrospective cohort	7	18	21	11	14	59.5	67.5
Wolter 1997^42^	Australia	RCT	High	13	18	39	28	22^b^	22^b^
Ibrahim 2017^33^	Australia	Prospective cohort	7	47	68	55.3	58.9	6.3	6.3
Yadev 2022^44^	Canada	Retrospective cohort	7	341	239	59.8	51.9	61.7	65
Ibrahim 2019^34^	Australia	RCT	High	89	91	60	47	7	7.2
Stovroff 1994^39^	USA	Prospective cohort	7	8	8	87.8	100	9.1	9
Pena 2013^46^	Chile	Prospective cohort	8	111	81	22.5	40.7	1.17	0.83

OPAT, outpatient parenteral antimicrobial therapy; IPAT, inpatient parenteral antimicrobial therapy; NR, not reported; RCT, randomized clinical trial; USA, United States of America.

^a^Newcastle–Ottawa Quality Assessment Scale for non-randomized studies and Cochrane risk of bias assessment tool for randomized studies.

^b^Median.

### Risk of bias

The summary of risk assessment for the included studies is presented in Table 1. One RCT was assessed as having some concern,^31^ while four RCTs were evaluated as having high concern.^27,34,42,43^ All other studies were assessed as high quality. Detailed quality assessments for the randomized and non-randomized studies is presented in Tables S4 and S5, respectively.

### Clinical characteristics

Table 2 presents the clinical characteristics and outcomes of the included studies. The primary disease conditions studied were cystic fibrosis,^29,33,42^ febrile neutropenia,^27,31,40^ infective endocarditis,^28,36^ cellulitis^34,35,44^ and appendicitis.^30,39,41^ Some studies did not report the specific interventions used to treat these conditions^31,32,35,37,43–45^ or the types of OPAT models employed.^30,36,38,41,45^

**Table 2.** Clinical characteristics and outcomes of patients receiving parenteral antimicrobial therapy

Author and year	Disease condition	OPAT model	Intervention		Health outcomes
			OPAT	IPAT	
Hendricks 2011^31^	Febrile neutropenia	H-OPAT	Aminoglycoside and vancomycin or ceftazidime alone; for allergic patients’ imipenem alone or an aztreonam-containing regimen	NR	Complication OPAT 9%, IPAT 8%; readmission OPAT 3 (9%); mortality 0%; patient-reported quality of life was similar between the two groups.
Hensey 2017^32^	Pyelonephritis, meningitis	H-OPAT	NR	NR	Unplanned readmission within 30 days pyelonephritis IPAT 8 (7%), OPAT 1 (8%); meningitis IPAT 2 (13%), OPAT 2 (7%)
Martel 1994^37^	Osteomyelitis, septic arthritis septic bursitis, cellulitis, cystic fibrosis, complicated urinary tract infection, severe external otitis, chronic sinusitis, cutaneous blastomycosis, endocarditis, lung abscess, liver abscess	S-OPAT	Penicillin G, cloxacillin, cephalothin, cefazolin, cefoxitin, cefotaxime, cefoperazone, ceftriaxone, ceftazidime, gentamicin, netilmicin, tobramycin, amikacin, clindamycin, erythromycin, gancyclovir, amphotericin B	NR	Treatment failure OPAT 2 (6.1%), IPAT 1 (5.9%); readmission OPAT 2 (6.1%); OPAT patients had a significantly higher internal locus of control, IPAT group had a significantly higher external locus of control.
Antoniskis 1978^28^	Infective endocarditis, osteomyelitis	S-OPAT	Methicillin, cephalothin, nafcillin, penicillin G, cefazolin, vancomycin, gentamicin, ampicillin	Methicillin, cephalothin, nafcillin, penicillin G, cefazolin, vancomycin, gentamicin, ampicillin	There are similar antibiotic-related complications; there is no infection of the intravenous cannula in either group. readmission OPAT 3 (23.1%)
Kameshwar 2016^35^	Cellulitis	H-OPAT	NR	NR	NR
Donati 1987^29^	Cystic fibrosis	H-OPAT, S-OPAT	Tobramycin, semisynthetic penicillin, cephalosporin	Tobramycin, semisynthetic penicillin, cephalosporin	No complications relating to the catheter; 65% of OPAT patients and 68% of IPAT patients required retreatment for pulmonary exacerbations; 85% of OPAT patients returned to their daily routines.
Warner 1998^41^	Acute appendicitis	NR	Cefotetan, gentamicin, ampicillin sodium/sulbactam sodium	Cefotetan, gentamicin, ampicillin sodium/sulbactam sodium	Negative appendectomy IPAT 12.3%, OPAT 9.2%; perforation IPAT 26.2%, OPAT 18.3%
Yong 2008^45^	Urinary tract infections, infective endocarditis, bone and joint infection, diabetic foot, septicaemia, orbital procedure, mouth, larynx or pharynx disorder	NR	NR	NR	Complication OPAT 18 (25%); cure OPAT 59 (81.9%), IPAT 75 (80.6%); readmission OPAT 13 (18.1%), IPAT 18 (19.4%); mortality OPAT 1 (1.4%), IPAT 1 (1.1%)
Ramasubramanian 2018^38^	Acute pyelonephritis	NR	Ertapenem	Ertapenem	NR
Wolter 2004^43^	Urinary tract infection, cellulitis, pneumonia, osteomyelitis, mycobacterial infection, cystic fibrosis, bronchiectasis	Home infusion	NR	NR	Adverse event OPAT 4 (11.4%), IPAT 4 (11.2%); readmission within 30 days OPAT 7 (15.9%), IPAT 4 (10.5%); no differences in improvements in quality of life between the two groups after treatment.
Ahmed 2007^27^	Febrile neutropenia	C-OPAT	Ceftriaxone plus amikacin	Imipenem	Adverse event OPAT 8 (13.1%), IPAT 4 (6.9%); favourable outcome OPAT 58 (95%), IPAT 56 (97%); mortality OPAT 2 (3.3%), IPAT 2 (3.4%)
Talcott 1994^40^	Febrile neutropenia	S-OPAT	Mezlocillin, gentamicin, ceftazidime, vancomycin	Mezlocillin, gentamicin, ceftazidime, vancomycin	Adverse events OPAT 4 (13.3%); readmission OPAT 9 (30%); quality of life improved during home therapy
Fishman 2000^30^	Perforated appendicitis	NR	Piperacillin-tazobactam, allergic patients gentamicin and clindamycin	Ampicillin, gentamicin, clindamycin	No mortality; readmission OPAT 4 (7.8%), IPAT 5 (5.1%); strong satisfaction with the home antibiotic regimen.
Lacroix 2014^36^	Infective endocarditis	NR	Oxacillin, vancomycin, ceftriaxone, amoxicillin, gentamycin, daptomycin, rifampicin	Oxacillin, vancomycin, ceftriaxone, amoxicillin, gentamycin, daptomycin, rifampicin	Adverse event OPAT 3 (16.7%); readmission OPAT 6 (33.3%), IPAT 7 (33.3%), death OPAT 1 (5.5%), IPAT 1 (4.8%)
Wolter 1997^42^	Cystic fibrosis	S-OPAT	Ceftazidime and tobramycin, imipenem used on allergy or failure	Ceftazidime and tobramycin, imipenem used on allergy or failure	No deaths, no short-term readmissions, no adverse events; no statistical difference in overall improvement in lung function; OPAT patients reported less disruption to their family life, personal life and sleeping pattern.
Ibrahim 2017^33^	Moderate/severe cellulitis	H-OPAT	Ceftriaxone	Flucloxacillin	Complication OPAT 3 (6%), IPAT 6 (10%); treatment failure OPAT 2 (4%), IPAT 8 (14%); readmission OPAT 0%, IPAT 1 (2%)
Yadev 2022^44^	Cellulitis	H-OPAT, C-OPAT	NR	NR	Adverse event OPAT 39 (11.4%); treatment failure OPAT 8 (2.3%)
Ibrahim 2019^34^	Cellulitis	H-OPAT, telemedicine	Ceftriaxone	Flucloxacillin	Readmission OPAT 2.25%; treatment failure OPAT 1 (1.1%), IPAT 7 (7.7%)
Stovroff 1994^39^	Ruptured appendicitis	S-OPAT	Ampicillin, gentamicin, clindamycin	Ampicillin, gentamicin, clindamycin	Complication 0%; readmission 0%; treatment failure 0%
Pena 2013^46^	Urinary tract infection	C-OPAT	Amikacin, ceftriaxone, cefotaxime	Amikacin, ceftriaxone, cefotaxime	OPAT 100% adherence, 0% readmission; efficacy OPAT 105 (100%), IPAT 72 (98.6%); adverse event OPAT 18 (16.2%), IPAT 58 (71.6%); mortality 0%

OPAT, outpatient parenteral antimicrobial therapy; IPAT, inpatient parenteral antimicrobial therapy; NR, not reported; S-OPAT, self or carer-administered OPAT at patients’ home; H-OPAT, physician or nurse-administered OPAT at patient home; C-OPAT, outpatient clinic or infusion centre administration.

### Economic evaluation

The economic evaluations of the included studies are presented in Table 3. The majority of the studies used a cost–consequence analysis.^27–33,36–43,45,46^ The cost perspective was specified in two-thirds of the studies,^28,29,31,33–36,39,40,42–45^ and half reported both the direct and indirect costs associated with parenteral antibiotic treatment.^31,33,34,36,39,40,42–44,46^ The average cost of treatment per episode of care was $10 453.67 in outpatient settings and $16 491.7 in inpatient settings. Notably, the average percentage of cost savings associated with OPAT was 36.11%.

**Table 3.** Economic evaluation of patients receiving parenteral antimicrobial therapy

Author and year	Economic analysis	Perspective	Type of cost	Mean cost (USD)		% of OPAT cost saving	Number of days of hospitalization avoided (mean)
				OPAT	IPAT		
Hendricks 2011^31^	CCA	Healthcare Payer’s	Direct and indirect	11 076.12	14 492.76	23.57	NR
Hensey 2017^32^	CCA	NR	NR	1371.12	1647.36	16.77	Polynephritis 1.5, meningitis 9^a^
Martel 1994^37^	CCA	NR	NR	7723.67	11 020.07	29.91	NR
Antoniskis 1978^28^	CCA	Hospital	NR	26 510.86	41 792.7	36.57	17.31
Kameshwar 2016^35^	CEA	Hospital	Direct and overhead	5205.88	4605.78	−13.03	5.6
Donati 1987^29^	CCA	Hospital	Direct	23 673.32	44 432.82	46.72	17.7
Warner 1998^41^	CCA	NR	NR	Non-perforated appendicitis 6561.01, perforated appendicitis 14 108.52	Non-perforated appendicitis 7385.20, perforated appendicitis 20 153.75		NR
Yong 2008^45^	CCA	Hospital	Direct	18 623.38^a^	18 136.45^a^	−2.68	24.3
Ramasubramanian 2018^38^	CCA	NR	NR	9373.89	19 516.23	51.97	NR
Wolter 2004^43^	CCA	Hospital	Direct and indirect	8461.92^a^	15 922.93^a^	46.86	6^a^
Ahmed 2007^27^	CCA	NR	NR	1878.03	2771.96	32.25	2.9
Talcott 1994^40^	CCA	NR	Direct and indirect	804.11	796.99	−0.89	5.3
Fishman 2000^30^	CCA	Hospital	NR	25 522.56	37 226.67	31.44	NR
Lacroix 2014^36^	CCA	Hospital	Direct and indirect	46 413.96	59 972.16	22.61	16.6
Wolter 1997^42^	CCA	Hospital	Direct and indirect	4747.45	9628.95	50.70	9^a^
Ibrahim 2017^33^	CCA	Hospital	Direct and indirect	1037.97	2309.17	55.05	3.7
Yadev 2022^44^	CBA	Healthcare Payer’s	Direct and indirect	2247.78	11 115.3	79.78	NR
Ibrahim 2019^34^	CEA	Societal	Direct and indirect	1672.27	3212.63	47.95	NR
Stovroff 1994^39^	CCA	Hospital	Direct and indirect	7663.52	12 754.49	39.92	5.1
Pena 2013^46^	CCA	NR	Direct and indirect	789.89	3062.94	74.21	3.2

OPAT, outpatient parenteral antimicrobial therapy; IPAT, inpatient parenteral antimicrobial therapy; NR, not reported; CBA, cost–benefit analysis; CCA: cost–consequence analysis; CEA, cost–effectiveness analysis; USD, US dollar.

^a^Median.

### Cost of parenteral antibiotic treatment

**Figure 2.**
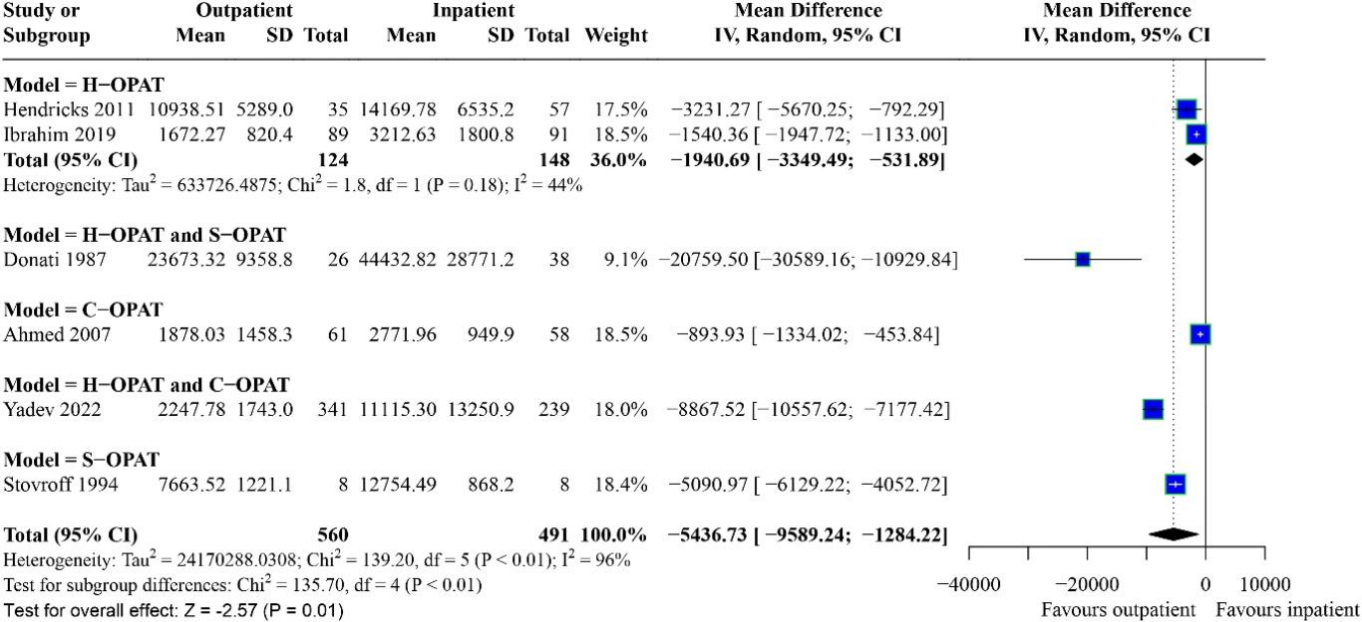
Cost of treatment: Forest plot comparison of outpatient and inpatient antimicrobial therapy. S-OPAT, self or carer-administered OPAT at patients’ home; H-OPAT, physician or nurse-administered OPAT at patient home; C-OPAT, outpatient clinic or infusion centre administration; IV, inverse variance; SD, standard deviation.

Six studies, involving three RCTs and three cohort studies, were included in the meta-analysis. The mean cost of OPAT per episode of care was $8012.24 (range: $1672.27–$23 673.32), and inpatient parenteral antimicrobial treatment was $14 742.83 (range: $2771.96–$44 432.82). The cost of parenteral antimicrobial treatment per episode of care was lower in the outpatient settings, with mean differences of −$5436.73 (95% CI: −$9589.24 to −$1284.22, I² = 96%; *P* = 0.01) compared to inpatient settings (Figure 2).

A sensitivity analysis was conducted on studies evaluating parenteral antimicrobial treatment’s direct and indirect costs, cost perspectives (payer), population (adult and paediatric), ways of hospitalization avoidance (admission avoidance and facilitation of discharge) and those published after 2000 (Figures S1–S9). The results confirmed those of the primary analysis (Figures S1–S3). The cost savings from the healthcare provider perspective favour OPAT, though the result is not statistically significant (Figure S4). Moreover, the cost savings of OPAT for the paediatric population, discharge facilitation and admission avoidance favour outpatient treatment, though the results are not statistically significant (Figures S7–S9).

## Discussion

To our knowledge, this is the first meta-analysis to assess the cost savings from OPAT compared to inpatient parenteral antimicrobial treatment. We conducted a quantitative analysis by inflating and adjusting the costs of parenteral antimicrobial treatment to 2023 US dollars. Parenteral antimicrobial treatment through OPAT programmes had a significantly lower cost compared to inpatient (hospital-bed based) treatment, with an estimated average cost saving of −$5436.73 per episode of care.

This finding of cost saving aligns with a previous review,^20^ although in that review, a robust estimate of the magnitude of cost saving was not possible due to the quality and methodological heterogeneity of the included studies, limiting direct comparisons. A gross estimate of the average cost saving associated with OPAT from the 35 heterogeneous studies they included, calculated from a ratio of average cost for OPAT to average cost for inpatient treatment, was 57.19% (−13.03% to 95.47%); in this review, the average percentage of cost savings calculated in the same way was 36.11%.

Most studies in the review examined indirect and direct costs, while only a few focused exclusively on direct costs. The cost savings from the OPAT programme are primarily attributed to the freeing up of hospital beds.^47^ However, a simple comparison of treatment costs between outpatient and inpatient settings does not provide a comprehensive assessment. A thorough evaluation should include all direct, indirect and intangible costs associated with treatment. Ultimately, the costs of parenteral antimicrobial treatment should be weighed against the benefits it offers to patients, healthcare providers, payers and the society.^48^

The cost–benefit of OPAT varies depending on the perspectives of patients, healthcare providers, payers and society. Despite the differing perspectives that lead to varied analyses and economic outcomes, from all viewpoints, the savings are primarily attributed to reductions in inpatient costs. However, in some healthcare systems, outpatient costs are typically borne directly by patients including co-payments for drugs and services, transportation to and from the healthcare facility, accommodation and home care services.^49^ These expenses may partially offset the savings from outpatient treatment.^47^ Nonetheless, the cost–benefit can be substantial when indirect benefits, such as increased productivity, savings on patient or carer transportation and accommodation costs, are taken into account.^48^

The substantial cost–benefit of the OPAT programme would mean that it can serve as an invaluable cost-saving tool for healthcare systems in the wake of increasing pressure to deliver high-quality patient-centred care while optimizing resources at a reduced cost, without compromising clinical outcomes.^50^ According to the World Health Organization, governments allocate between half and two-thirds of their health expenditures to hospital care. Alarmingly, nearly $300 billion is wasted annually due to hospital-related inefficiencies.^51^ Therefore, wider implementation of OPAT is likely to contribute to efficient resource/funding relocation, enhancing the overall capacity of the healthcare system. The OPAT programme improves patient flow and increases hospital bed capacity by allowing patients to receive treatment closer to home, thereby enabling hospitals to focus on acute medical conditions.^52^

Treatment in OPAT is both safe and effective compared to inpatient treatment.^10^ OPAT enables the treatment of patients infected with antimicrobial-resistant organisms. The risk of acquiring nosocomial infections with such organisms is low, thereby avoiding unplanned huge treatment costs associated with such infections.^53^ Beyond financial gains, it offers clinical benefits by improving patient’s quality of life through home-based treatment, which minimizes stress and disruptions to daily routines. It also promotes emotional well-being and allows for flexible scheduling, making the treatment experience more manageable and supportive.^14^

Despite the multiple benefits OPAT offers, it also has limitations. Some patients encountered difficulties in administering medications due to a lack of confidence in their skills,^17,54^ which could lead to complications related to administration devices.^55^ Others experienced challenges with showering, walking and sleeping during continuous intravenous drug administration,^56^ impacting their social activities^54^ and school attendance.^52^ A few families perceived OPAT as increasing their workload.^57^ Additionally, patients with limited transportation or those living far from healthcare facilities may need to travel for monitoring and medication administration.^58^

The strengths of this review include adherence to PRISMA guidelines and a comprehensive search across multiple databases, ensuring a robust methodology. Moreover, the review included studies that compared the actual costs associated with OPAT to those of inpatient treatment, with costs inflated and adjusted to a common currency and year for accurate comparison. However, there are notable limitations among the included studies. First, most studies were cohort studies, with only six being RCTs, and these RCTs were assessed as having a high risk of bias. Additionally, there was heterogeneity among the studies, which arose from their diverse healthcare system contexts. Second, over half of the studies reported limited cost data, which precluded their inclusion in the meta-analysis. Furthermore, publication bias could not be assessed due to the small number of included studies. Third, the included studies demonstrated heterogeneity in the disease conditions evaluated, with many focusing on uncommon indications for OPAT. Fourth, the included studies reported different ways of hospitalization avoidance (early discharge and admission avoidance) and patient populations (adult and paediatric). Fifth, the types of costs used to calculate the costs of parenteral antimicrobial treatment varied widely. However, a sensitivity analysis did not alter the primary results. Sixth, cost data in most of the included studies were evaluated as secondary outcomes. Furthermore, most studies did not specify the cost perspective, and those that did were limited to the hospital perspective. Consequently, pharmacoeconomic data comparing the costs of parenteral antimicrobial treatment in outpatient versus inpatient settings are scarce. Therefore, we recommend conducting more robust pharmacoeconomic studies that compare outpatient and inpatient parenteral antimicrobial treatment costs, incorporating all cost types from the perspective of patients, society, the healthcare system and payers. Moreover, studies comparing the cost of OPAT to complex outpatient oral antimicrobial therapy are warranted.

### Conclusions

Parenteral antimicrobial treatment through OPAT programmes significantly saves costs compared to inpatient settings from the payer perspective. For a comprehensive comparison of treatment costs in both settings, further pharmacoeconomic studies that include all types of treatment costs are essential. We recommend conducting studies that evaluate the costs of parenteral antimicrobial treatment from the perspectives of patients, healthcare providers, payers and the society.

## Supplementary Material

dlaf049_Supplementary_Data

## Supplementary data

Figures S1–S9 and Tables S1–S5 are available as Supplementary data at *JAC-AMR* Online.
