# Supplementary material for: Quantifying cost savings from outpatient parenteral antimicrobial therapy programme: a systematic review and meta-analysis
Source: JAC Antimicrob Resist. 2025 Apr 8;7(2):dlaf049. doi: 10.1093/jacamr/dlaf049 (PMC11976721; doi:10.1093/jacamr/dlaf049)
Supplement: dlaf049_Supplementary_Data [file dlaf049_supplementary_data.docx]

Table S1. Search strategy

| **Pubmed**  #1 Home[Title/Abstract] 299,683  #2 Domiciliary[Title/Abstract] 2,960  #3 Ambulatory[Title/Abstract] 94,768  #4 Ambulatory Care"[MeSH Terms] 57,214  #5 Out-patient*[Title/Abstract] 18,362  #6 Outpatient*[Title/Abstract] 238,788  #7 "Outpatients"[Mesh] 22,936  #8 Discharge[Title/Abstract] 260,002  #9 #1 OR #2 OR #3 OR #4 OR #5 OR #6 OR #7 858,233  #10 ( "Anti-Infective Agents/administration and dosage"[Mesh] ) 109,763  #11 "Antibiotic* therapy"[Title/Abstract] 42,789  #12 "Antibiotic* treatment"[Title/Abstract] 30,896  #13 "Antimicrobial therapy"[Title/Abstract] 15,973  #14 "Ambulatory management"[Title/Abstract] 398  #15 "Antibiotic* management"[Title/Abstract] 887  #16 #9 OR #10 OR #11 OR #12 OR #13 OR #14 187,403  #17 #8 AND #15 10,796  #18 Cost*[Title/Abstract] 860,165  #19 Economi*[Title/Abstract] 433,762  #20 Expen*[Title/Abstract] 201,572  #21 #19 OR #20 OR #21 1,325,371  #22 #17 AND #21 1,468 |
| --- |
| **Cochrane library (1991–August 22 2024)**  #1 (home):ti,ab,kw (Word variations have been searched) 64,910  #2 (domiciliary):ti,ab,kw (Word variations have been searched) 563  #3 (ambulatory):ti,ab,kw (Word variations have been searched) 26,559  #4 (outpatient*):ti,ab,kw (Word variations have been searched) 54,177  #5 (out-patient*):ti,ab,kw (Word variations have been searched) 5,111  #6 MeSH descriptor: [Outpatients] this term only 2,007  #7 (discharge):ti,ab,kw (Word variations have been searched) 52,567  #8 MeSH descriptor: [Ambulatory Care] this term only 3,914  #9 #1 OR #2 OR #3 OR #4 OR #5 OR #6 OR #7 OR #8 175,758  #10 MeSH descriptor: [Anti-Infective Agents] this term only 3,326  #11 (antibiotic* NEXT therapy):ti,ab,kw (Word variations have been searched) 7,149  #12 (antibiotic* NEXT treatment):ti,ab,kw (Word variations have been searched) 4,013  #13 (antibiotic* NEXT management):ti,ab,kw (Word variations have been searched) 86  #14 (ambulatory NEXT management):ti,ab,kw (Word variations have been searched) 64  #15 (antimicrobial NEXT therapy):ti,ab,kw (Word variations have been searched) 1,430  #16 #10 OR #11 OR #12 OR #13 OR #14 OR #15 13,806  #17 #9 AND #16 1,934  #18 (cost*):ti,ab,kw (Word variations have been searched) 95,570  #19 (Economi*):ti,ab,kw (Word variations have been searched) 40,471  #20 (Expen*):ti,ab,kw (Word variations have been searched) 19,190  #21 #18 OR #19 OR #20 123,549  #22 #17 AND #21 421 |
| **Ovid MEDLINE(R) <1946 to August 20 2024>**  #1 home.tw. 295,474  #2 domiciliary.tw. 2,905  #3 ambulatory.tw. 92,328  #4 *Ambulatory Care/ 20,968  #5 outpatient*.tw. 222,585  #6 *out-patient*.tw. 18,111  #7 Outpatients/ 7,742  #8 discharge.tw. 258,574  #9 #1 or #2 or #3 or #4 or #5 or #6 or #7 or #8 685,236  #10 Anti-Bacterial Agents/ or Anti-Infective Agents/ 834,429  #11 "antibiotic*therapy".tw. 53  #12 "antibiotic* treatment".tw. 30,594  #13 "antimicrobial therapy".tw. 15,638  #14 "ambulatory management".tw. 386  #15 "antibiotic* management".tw. 797  #16 #10 or #11 or #12 or #13 or #14 or #15 500,362  #17 #9 and #16 16,295  #18 cost*.tw. 845,405  #19 Economi*.tw. 408,431  #20 Expen*.tw. 200,392  #21 #18 or #19 or #20 1,292,023  #22 #17 and #21 1935 |
| **Embase (ELSEVIER, 1947–August 22 2024)**  #1 home:ti,ab,kw 432,488  #2 domiciliary:ti,ab,kw 4,299  #3 ambulatory:ti,ab,kw 141,894  #4 'ambulatory care'/exp 58,208  #5 outpatient*:ti,ab,kw 398,951  #6 out-patient*:ti,ab,kw 32,825  #7 'outpatient'/exp 170,433  #8 discharge:ti,ab,kw 404,907  #9 #1 OR #2 OR #3 OR #4 OR #5 OR #6 OR #7 OR #8 1,311,593  #10 'antibiotic agent':ti,ab,kw 817  #11 'anti-infective agent':ti,ab,kw 276  #12 'antibiotic* therapy':ti,ab,kw 60,897  #13 'antibiotic* treatment':ti,ab,kw 43,564  #14 'antimicrobial therapy':ti,ab,kw 22,227  #15 'ambulatory management':ti,ab,kw 705  #16 'antibiotic* management':ti,ab,kw 1,306  #17 #10 OR #11 OR #12 OR #13 OR #14 OR #15 OR #16 120,352  #18 #9 AND #17 12,575  #19 cost*:ti,ab.kw 1,148,650  #20 economi*:ti,ab.kw 513,507  #21 expen*:ti,ab.kw 271,531  #22 #19 OR #20 OR #21 1,694,821  #23 #18 AND #22 1,737 |
| **Web of Sciences**  #1 TS=(home) 620,926  #2 TS=(domiciliary) 3,225  #3 TS=(ambulatory) 102,734  #4 TS=(outpatient*) 236,502  #5 TS=(out-patient*) 15,991  #6 TS=(discharge) 770,518  #7 #1 OR #2 OR #3 OR #4 OR #5 OR #6 1,668,750  #8 TS=("antibiotic* therapy") 39,591  #9 TS=("antibiotic* treatment") 31,494  #10 TS=("antimicrobial therapy") 17,542  #11 TS=("ambulatory management") 395  #12 TS=("antibiotic* management") 880  #13 TS=("antibacterial agent") 7,539  #14 TS=("anti-infective agent") 212  #15 #8 OR #9 OR #10 OR #11 OR #12 OR #13 OR #13 89,603  #16 #7 AND #15 8,842  #17 TS=(cost*) 2,643,981  #18 TS=(economi*) 1,890,562  #19 TS=(expen*) 496,358  #20 #17 OR #18 OR #19 4,518,733  #21 #16 AND #20 1244 |

Table S2. Detailed calculation of OPAT and inpatient costs

| Author and year | Currency, price year | OPAT cost per patient | | IPAT cost per patient | | Number of days of hospitalisation avoided |
| --- | --- | --- | --- | --- | --- | --- |
|  |  | Primary | Estimated by SRMA authors | Primary | Estimated by SRMA authors |  |
| Hendricks 2011 | USD, 2008 | Mean direct cost 7830, mean indirect cost 98.5 | Total cost= direct cost + indirect cost 7830+98.5 =7928.5 | Mean direct cost 10143 4678, indirect cost 231.2 | Total cost= direct cost + indirect 10143+231.2 =10374.2 |  |
| Hensey 2017 | AUD, 2013 | Pyelonephritis=5040 Meningitis=58380 | Total cost = Pyelonephritis + Meningitis= 63420 mean cost 63420/41=1546.83 | Pyelonephritis=19200 Meningitis=222400 | Total cost = Pyelonephritis + Meningitis= 241600 mean cost 241600/130=1858.46 | Median 1.5 for polynephritis, 9 for meningitis |
| Martel 1994 | USD, 1991 | Mean cost $3852, range$2085 to $7794 |  | Mean cost $5493, range $1490 to $872 |  |  |
| Antoniskis 1978 | USD, 1976 | Mean cost 6357.52 |  | Mean cost 10022.23 |  | Mean 17.31 |
| Kameshwar 2016 | AUD, 2013 | mean cost AU$5873 (95% CI: AU$5212–AU$6534) |  | Mean cost 5196 (95% CI: AU$4567–AU$5824) |  | Mean 5.6 |
| Donati 1987 | USD, 1986 | Mean cost 9985, SEM 774 |  | Mean cost 18741, SEM 1970 |  | Mean 17.7 |
| Warner 1998 | USD, 1996 | Non-perforated Appendicitis mean cost = $3,638, SD = $1,633; Perforated Appendicitis mean cost $7,823, SD = $2,366 |  | Non-perforated Appendicitis mean costs= $4,095, SD = $1,280; Perforated Appendicitis mean cost $11,175, SD = $3,893 |  |  |
| Yong 2008 | USD, 2006 | Mean cost 12736, median 8863, range 196 to 97578 |  | Mean cost 12403, median 3910, range 736–388758 |  | Mean 24.3 |
| Ramasubramanian 2018 | India, 2014 | Mean charges at Apollo hospital/OPAT (INR 203268), OPAT only (INR 37497), KIMS hospital/OPAT (INR 87103), OPAT only (INR 21589) | Total cost = Apollo hospital/OPAT + OPAT only = (41x203268) +(22x37497) + (22x87103) + (4x21589)  mean cost = 11161544/89= 125410.6 | Mean charges at Apollo (INR 340870), KIMS 161406) | Total cost = Apollo hospital only = KIMS hospital only = (5x340870) + (4x161406) = 2349974 mean cost = 2349974/9= 261102.2 |  |
| Wolter 2004 | Pound, 2003 | Median cost 3686, range 600 to 32612 |  | Median cost 6936, range 2816 to 28160 |  | Median 6 days |
| Ahmed 2007 | USD, 2004 | Mean 1,208, SD 93 |  | Mean 1,783, SD 611 |  | Mean 2.9 days |
| Talcott 1994 | USD, 1991 | Total cost 12,031 | Mean cost = 12031/30=401.03 | Total cost = 10732 | Mean cost = 10732/27=397.48 | Median 5.3 days |
| Fishman 2000 | USD, 1999 | Mean hospital charges $13,188, home charge 1575 | Total charge = hospital + home charge = 13188+1575= 14763 | Mean $21,533 |  |  |
| Lacroix 2014 | Euro, 2012 |  | Mean cost per OPAT patient (outpatient treatment cost) = 67,943 euros/18 = 3774.6, Total hospitalization cost for 18 patients=1,125 euros/day×23.5 days×18 = 26437.5, total mean cost = 26437.5+3774.6 = 30212.1 |  | Daily hospitalization cost = 335,250 euros/298 days = 1125 Mean cost per hospital patient= 1125x34.7 = 39037.5  ​ | Total 298 days saved mean = 298/18= 16.6days |
| Wolter 1997 | USD, 1993 | Mean cost 2476 |  | Mean cost 5028 |  | median 9 days |
| Ibrahim 2017 | AUD, 2015 | Total cost 93 280, mean cost 1166 |  | Total cost 228 720, mean cost 2594 |  | Total 176 days mean 176/47 = 3.7 days |
| Yadev 2022 | Canada, 2015 | Mean 2164, SD 1678 |  | Mean 10701, SD 12757 |  |  |
| Ibrahim 2019 | AUD, 2017 | Mean 1965, SD 964 |  | Mean 3775, SD 2116 |  |  |
| Stovroff 1994 | USD, 1991 | Mean 3822, SD 609 |  | Mean 6361, SD 433 |  | Mean 5.14 |
| Pena 2013 | Chile, 2011 |  | Direct cost =170,928, mean indirect cost =12,704 Total cost =183,632 |  | Direct cost =696,790, mean indirect cost =15,275 Total cost =712,065 | Mean 3.2 |

OPAT: Outpatient parenteral antimicrobial therapy, IPAT: Inpatient parenteral antimicrobial therapy, USD: United States Dollar, AUD: Australia Dollar, SD: Standard deviation

Table S3. Full-text articles excluded

|  | Study | **Reasons for exclusion** |
| --- | --- | --- |
| 1 | Chamberlain TM, Lehman ME, Groh MJ, Munroe WP, Reinders TP. Cost analysis of a home intravenous antibiotic program. American Journal of Hospital Pharmacy. 1988 Nov 1;45(11):2341-5. | Not comparative study |
| 2 | Ibrahim LF, Hopper SM, Babl FE, Bryant PA. Who can have parenteral antibiotics at home?: a prospective observational study in children with moderate/severe cellulitis. The Pediatric Infectious Disease Journal. 2016 Mar 1;35(3):269-74. | Simulation |
| 3 | Rodríguez-Cerrillo M, Poza-Montoro A, Fernandez-Diaz E, Matesanz-David M, Romero AI. Treatment of elderly patients with uncomplicated diverticulitis, even with comorbidity, at home. European journal of internal medicine. 2013 Jul 1;24(5):430-2. | No distinction between oral and parenteral outpatient antibiotic therapy |
| 4 | González-Ramallo VJ, Mirón-Rubio M, Mujal A, Estrada O, Forné C, Aragón B, Rivera AJ. Costs of outpatient parenteral antimicrobial therapy (OPAT) administered by Hospital at Home units in Spain. International journal of antimicrobial agents. 2017 Jul 1;50(1):114-8. | Not comparative study |
| 5 | Psaltikidis EM, Silva EN, Moretti ML, Trabasso P, Stucchi RS, Aoki FH, Cardoso LG, Höfling CC, Bachur LF, Ponchet DD, Colombrini MR. Cost-utility analysis of outpatient parenteral antimicrobial therapy (OPAT) in the Brazilian national health system. Expert review of pharmacoeconomics & outcomes research. 2019 May 4;19(3):341-52. | Simulation |
| 6 | Dimitrova M, Gilchrist M, Seaton RA. Outpatient parenteral antimicrobial therapy (OPAT) versus inpatient care in the UK: a health economic assessment for six key diagnoses. BMJ open. 2021 Sep 1;11(9):e049733. | Simulation |
| 7 | Safrin S, Siegel D, Black D. Pyelonephritis in adult women: inpatient versus outpatient therapy. The American journal of medicine. 1988 Dec 1;85(6):793-8. | No distinction between oral and parenteral outpatient antibiotic therapy |
| 8 | Del Prete SA, Ryan SP, Jacobson JS, Erichson RB, Weinstein PL, Grann VR. Safety and costs of treating neutropenic fever in an outpatient setting. Connecticut Medicine. 1999 Dec 1;63(12):713-7. | No distinction between IPAT and OPAT |
| 9 | Chrysochoou EA, Hatziagorou E, Kirvassilis F, Tsanakas J. Home intravenous antibiotic therapy in children with cystic fibrosis: clinical outcome, quality of life and economic benefit. Hippokratia. 2016 Oct;20(4):279. | Not comparative study |
| 10 | Wilimas JA, Flynn PM, Harris S, Day SW, Smith R, Chesney PJ, Rodman JH, Eguiguren JM, Fairclough DL, Wang WC. A randomized study of outpatient treatment with ceftriaxone for selected febrile children with sickle cell disease. New England Journal of Medicine. 1993 Aug 12;329(7):472-6. | Not OPAT study |
| 11 | Williams B, Muklewicz J, Steuber TD, Williams A, Edwards J. Comparison of inpatient standard-of-care to outpatient oritavancin therapy for patients with acute uncomplicated cellulitis. Journal of Pharmacy Practice. 2023 Feb;36(1):27-32. | No distinction between oral and parenteral outpatient antibiotic therapy |
| 12 | Giron RM, Cisneros C, Nakeeb ZA, Hoyos N, Martinez C, Ancochea J. Efficiency of the home intravenous antibiotics treatment in cystic fibrosis. Medicina clínica. 2006 Oct 1;127(15):567-71. | Not comparative study |
| 13 | Craven PC. Treating bone and joint infections with teicoplanin: hospitalization vs outpatient cost issues. Hospital formulary. 1993 Jan 1;28:41-5. | Not OPAT study |
| 14 | Krauth C, Busse R, Smaczny C, Ullrich G, Wagner TO, Weber J, Welte T. Cost analysis of hospital and home iv Therapy in adults with cystic fibrosis. Results of a prospective controlled study: Ergebnisse einer prospektiven kontrollierten Studie. Medizinische Klinik. 1999 Oct;94:541-8. | No distinction between IPAT and OPAT |
| 15 | Elliott RA, Thornton J, Webb AK, Dodd M, Tully MP. Comparing costs of home-versus hospital-based treatment of infections in adults in a specialist cystic fibrosis center. International journal of technology assessment in health care. 2005 Oct;21(4):506-10. | Not OPAT study |
| 16 | Robinson PD, Vaughan S, Missaghi B, Meatherall B, Pattullo A, Kuhn S, Conly J. A case series of infectious complications in medical tourists requiring hospital admission or outpatient home parenteral therapy. Official Journal of the Association of Medical Microbiology and Infectious Disease Canada. 2022 Mar 1;7(1):64-74. | No distinction between IPAT and OPAT |
| 17 | Debaenst N, Seyler L, Putman K, Van Den Borre I, Vanlauwe J. Outpatient parenteral antibiotic therapy for the treatment of prosthetic knee and hip infections in Belgium, a cost minimization analysis. Acta Clinica Belgica. 2022 Jan 2;77(1):10-7. | Simulation |
| 18 | Wilimas JA, Flynn PM, Harris S, Day SW, Smith R, Chesney PJ, Rodman JH, Eguiguren JM, Fairclough DL, Wang WC. A randomized study of outpatient treatment with ceftriaxone for selected febrile children with sickle cell disease. New England Journal of Medicine. 1993 Aug 12;329(7):472-6. | Not OPAT study |
| 19 | Pena DA, Viviani ST, Le Corre PN, Morales MV, Montecinos BC, Gajardo SC. Treatment of urinary tract infections in febrile infants: experience of outpatient intravenous antibiotic treatment. Revista Chilena de Infectologia: Organo Oficial de la Sociedad Chilena de Infectologia. 2009 Aug 1;26(4):350-4. | Simulation |
| 20 | Greiner W, Klettke U, Wahn U. Economic aspects in treatment of cystic fibrosis with chronic pulmonary pseudomonas infection. Ambulatory intravenous therapy in comparison with inpatient treatment. Medizinische Klinik (Munich, Germany: 1983). 1997 Oct 1;92(10):626-9. | Cost data can not be extracted |
| 21 | Eaton EF, Mathews RE, Lane PS, Paddock CS, Rodriguez JM, Taylor BB, Saag MS, Kilgore ML, Lee RA. A 9-point risk assessment for patients who inject drugs and require intravenous antibiotics: focusing inpatient resources on patients at greatest risk of ongoing drug use. Clinical Infectious Diseases. 2019 Mar 5;68(6):1041-3. | Not OPAT study |
| 22 | Staples JA, Ho M, Ferris D, Hayek J, Liu G, Tran KC, Sutherland JM. Outpatient versus inpatient intravenous antimicrobial therapy: a population-based observational cohort study of adverse events and costs. Clinical Infectious Diseases. 2022 Dec 1;75(11):1921-9. | Simulation |
| 23 | Thornton J, Elliott RA, Tully MP, Dodd M, Webb AK. Clinical and economic choices in the treatment of respiratory infections in cystic fibrosis: comparing hospital and home care. Journal of Cystic Fibrosis. 2005 Dec 1;4(4):239-47. | Not OPAT study |
| 24 | Santolaya ME, Alvarez AM, Avilés CL, Becker A, Cofré J, Cumsille MA, O'Ryan ML, Payá E, Salgado C, Silva P, Tordecilla J. Early hospital discharge followed by outpatient management versus continued hospitalization of children with cancer, fever, and neutropenia at low risk for invasive bacterial infection. Journal of Clinical Oncology. 2004 Sep 15;22(18):3784-9. | No distinction between oral and parenteral outpatient antibiotic therapy |
| 25 | Raisch DW, Holdsworth MT, Winter SS, Hutter JJ, Graham ML. Economic comparison of home-care-based versus hospital-based treatment of chemotherapy-induced febrile neutropenia in children. Value in Health. 2003 Mar 1;6(2):158-66. | No distinction between IPAT and OPAT |
| 26 | Lavie M, Vilozni D, Sokol G, Somech R, Szeinberg A, Efrati O. Hospital versus home treatment of respiratory exacerbations in cystic fibrosis. Medical science monitor: international medical journal of experimental and clinical research. 2011;17(12):CR698. | Cost data can not be extracted |
| 27 | Board N, Brennan N, Caplan GA. A randomised controlled trial of the costs of hospital as compared with hospital in the home for acute medical patients. Australian and New Zealand journal of public health. 2000 Jun;24(3):305-11. | Not OPAT study |

Table S4. Quality assessment of randomised studies

| Author (year) | Random sequence generation (selection bias) | Allocation concealment (selection bias) | Blinding (performance and detection bias) | Incomplete outcome data (attrition bias) | Selective reporting (reporting bias) | Overall |
| --- | --- | --- | --- | --- | --- | --- |
| Ahmed, 2007 | Unclear risk Reason: the method of sequence generation was not reported. | Unclear risk Reason: the method for allocation concealment was not reported. | High-risk Reason: Study participants were not blinded. | Low-risk Reason: loss of 7.6% (5/66) in the intervention group and 7.9% (5/63) in the comparator group. | Low-risk Reason: all data included. | High risk |
| Ibrahim, 2019 | Low-risk Reason: the randomization web-based randomization tool and stratified by disease and age. | Low-risk Reason: "Preparation of the randomization sequence was completed by a data management coordinator who was independent of the research team and had no further role in the trial." | High-risk Reason: Study participants were not blinded. | Low-risk Reason: loss of 4.3% (4/93) in the intervention group and 4.2% (4/95) in the comparator group. | Low-risk Reason: all data included. | High risk |
| Hendricks, 2011 | Low-risk Reason: Random assignments were computer-generated using blocks and stratified using colony-stimulating factors. | Low-risk Reason: sequenced sealed envelopes were used. | Unclear risk Reason: the method for blinding was not reported. | Low-risk Reason: 6% (3/50) loss in the intervention group and 7% (5/71) in the comparator group. | Low-risk Reason: all data included. | Some concerns |
| Wolter, 1997 | High-risk Reason: Subjects experiencing recurrent episodes automatically alternated treatment arms after initial randomization. | High-risk Reason: despite the use of a sequenced sealed envelope, the alternation of participants experiencing recurrent episodes after initial randomization makes concealment difficult. | Unclear risk Reason: the method for blinding was not reported. | Low-risk Reason: Complete data reported. | Low-risk Reason: all data included. | High risk |
| Wolter, 2004 | Low-risk Reason: "Patients were randomized independently by random numbers generated by the hospital pharmacy." | Unclear risk Reason: the method for allocation concealment was not reported. | Unclear risk Reason: the method for blinding was not reported. | High-risk Reason: high attrition "After randomization, four (9.1%) individuals withdrew from the home therapy group, two preferring to stay in the hospital. There were nine (23.7%) withdrawals from the hospital group, eight individuals preferring an alternative home treatment option offered by the ward outside the trial setting." | Low-risk Reason: all data included. | High risk |

Table S5. Quality assessment of non-randomised studies

| Author | Selection | Comparability | Exposure |
| --- | --- | --- | --- |
| Hensey 2017 | **** | * | *** |
| Martel 1994 | *** | ** | *** |
| Antoniskis 1978 | **** | ** | *** |
| Kameshwar 2016 | **** | * | *** |
| Donati 1987 | **** | ** | *** |
| Warner 1998 | **** | ** | ** |
| Yong 2008 | **** | ** | *** |
| Ramasubramanian 2018 | **** | ** | ** |
| Talcott 1994 | **** | * | ** |
| Fishman 2000 | **** | * | *** |
| Lacroix 2014 | **** | * | ** |
| Ibrahim 2017 | **** | * | ** |
| Yadev 2022 | **** | * | ** |
| Stovroff 1994 | **** | * | ** |
| Pena 2013 | **** | * | *** |

Figure S1: Cost of treatment: Forest plot comparison of outpatient and inpatient antimicrobial therapy by disease condition


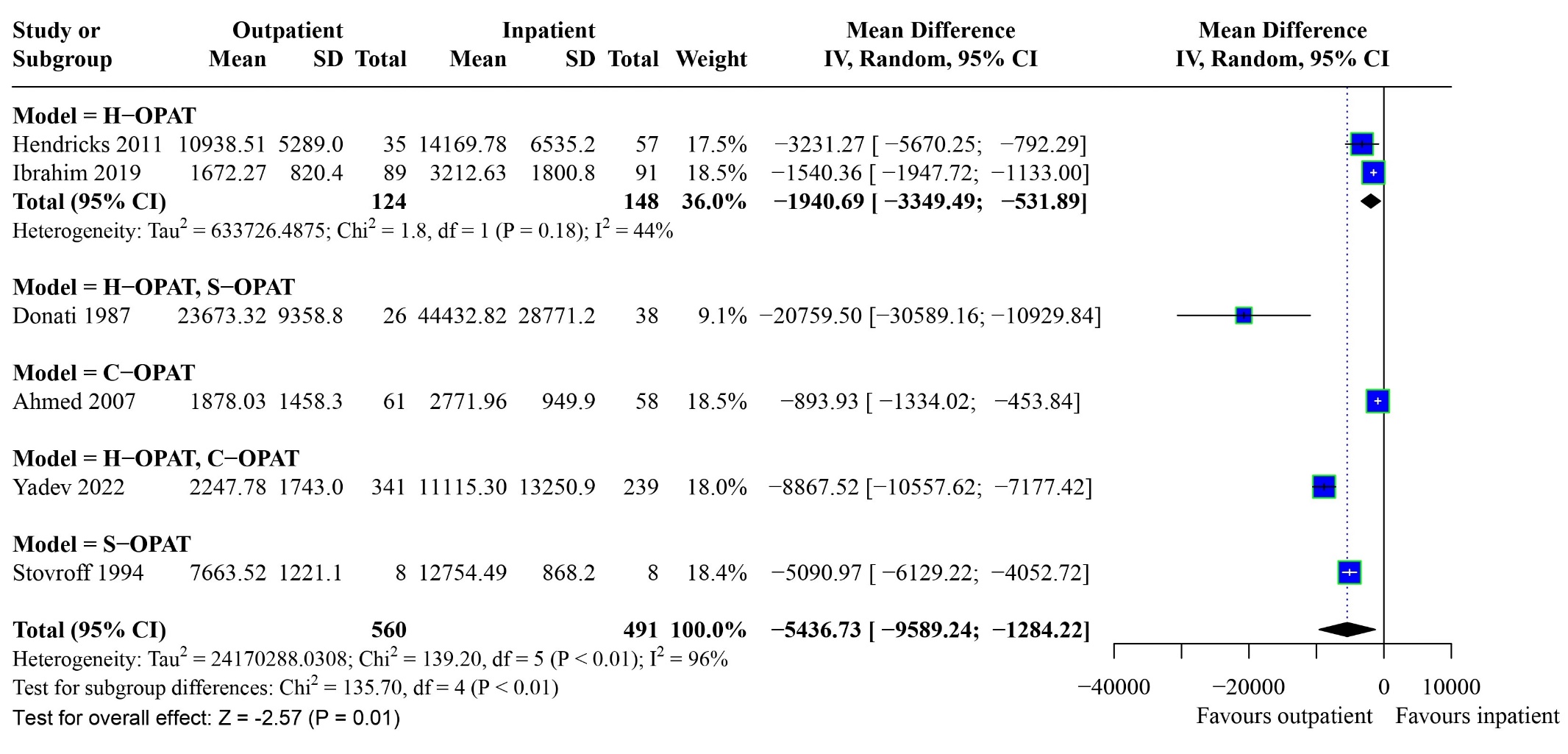
S-OPAT, self or carer-administered OPAT at patients’ home; H-OPAT, physician or nurse-administered OPAT at patient home; C-OPAT, outpatient clinic or infusion centre administration, IV, Inverse Variance; SD, Standard Deviation

Figure S2: Cost of treatment: Forest plot comparison of outpatient and inpatient antimicrobial therapy by study year


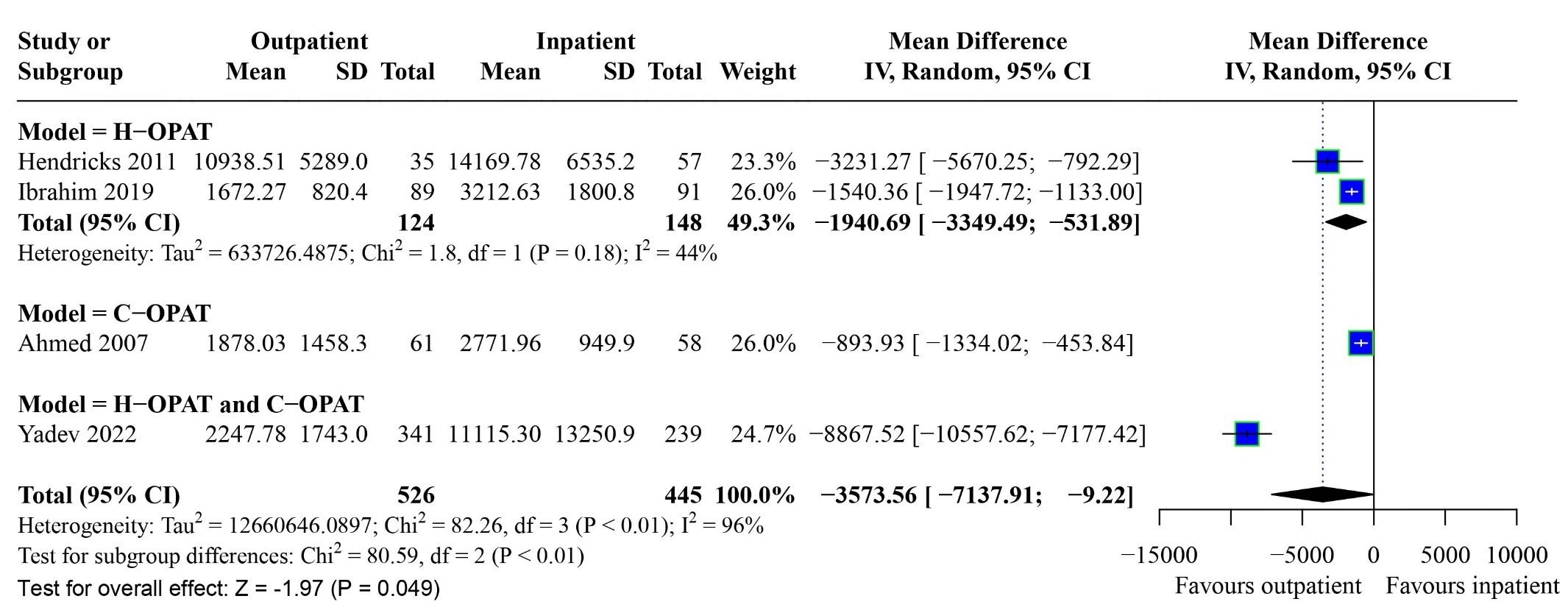
S-OPAT, self or carer-administered OPAT at patients’ home; H-OPAT, physician or nurse-administered OPAT at patient home; C-OPAT, outpatient clinic or infusion centre administration, IV, Inverse Variance; SD, Standard Deviation

Figure S3: Cost of treatment: Forest plot comparison of outpatient and inpatient antimicrobial therapy by payer perspective


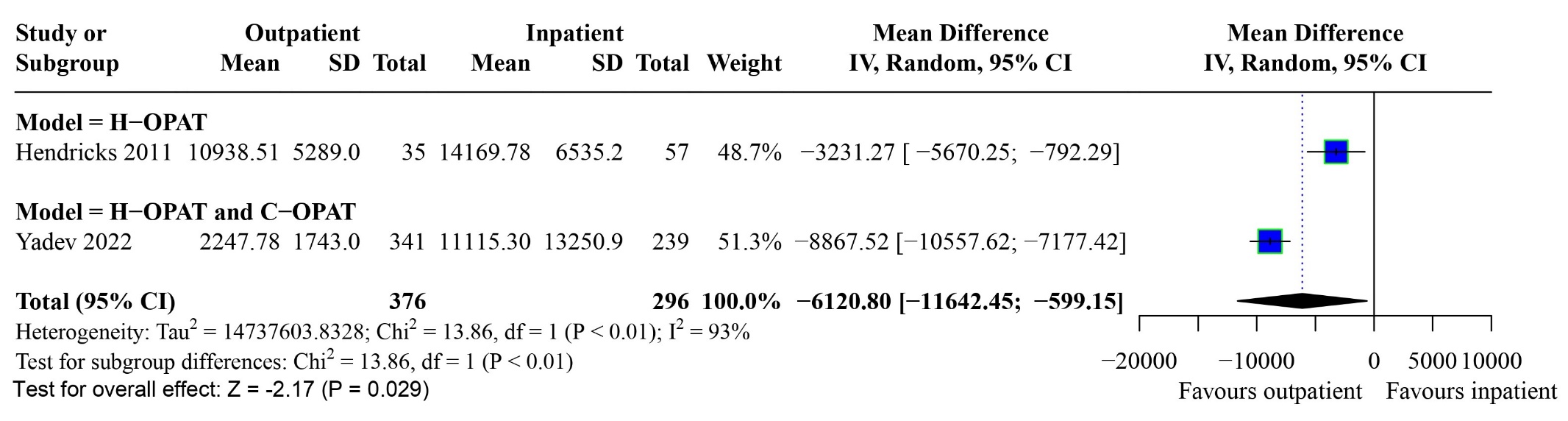


H-OPAT, physician or nurse-administered OPAT at patient home; C-OPAT, outpatient clinic or infusion centre administration, IV, Inverse Variance; SD, Standard Deviation

Figure S4: Cost of treatment: Forest plot comparison of outpatient and inpatient antimicrobial therapy from a healthcare provider perspective


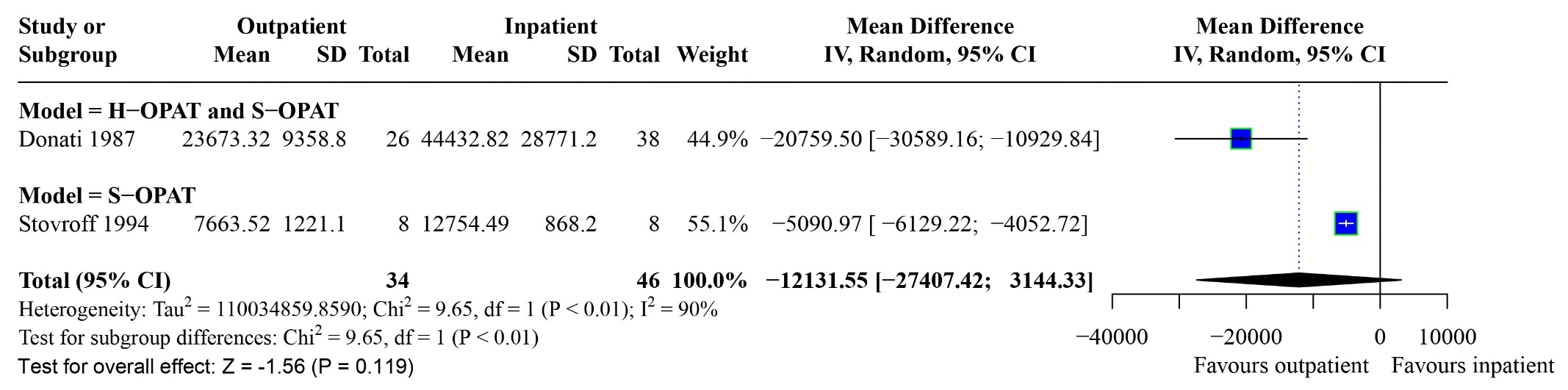


S-OPAT, self or carer-administered OPAT at patients’ home; H-OPAT, physician or nurse-administered OPAT at patient home; IV, Inverse Variance; SD, Standard Deviation

Figure S5: Cost of treatment: Forest plot comparison of outpatient and inpatient antimicrobial therapy from a societal perspective


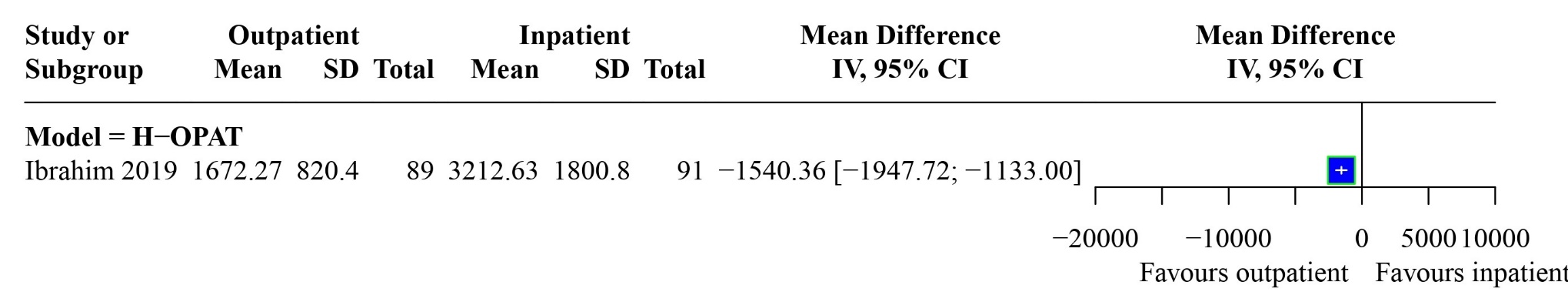


H-OPAT, physician or nurse-administered OPAT at patient home; IV, Inverse Variance; SD, Standard Deviation

Figure S6: Cost of treatment: Forest plot comparison of outpatient and inpatient antimicrobial therapy by adult population


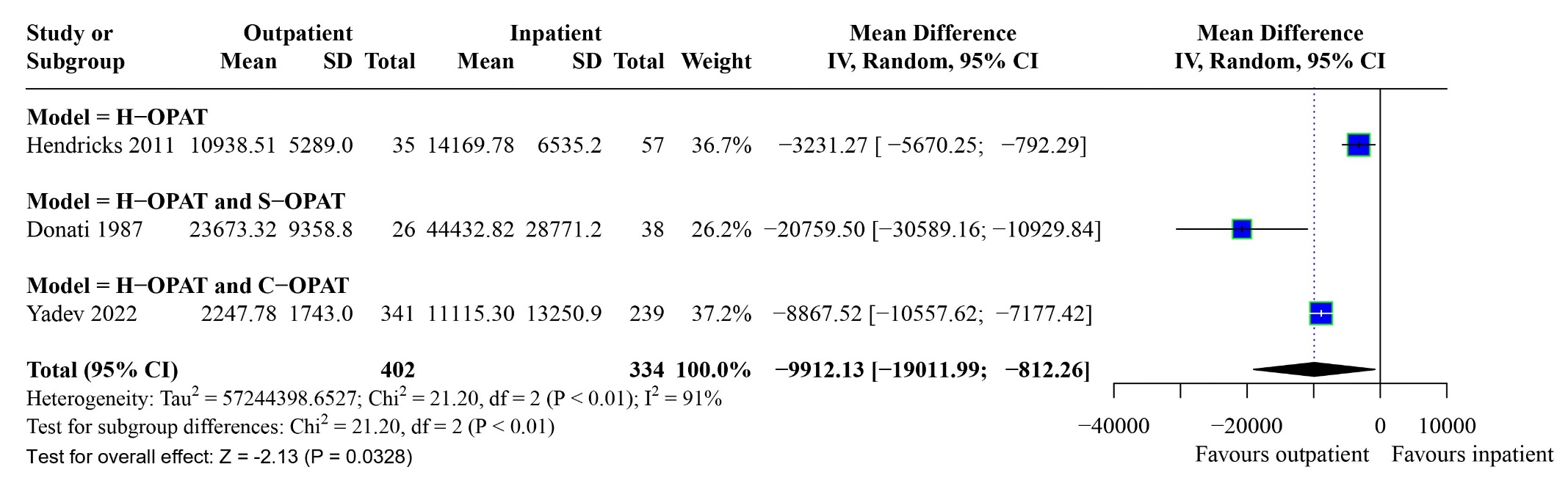
C-OPAT, outpatient clinic or infusion centre administration, S-OPAT, self or carer-administered OPAT at patients’ home; H-OPAT, physician or nurse-administered OPAT at patient home; IV, Inverse Variance; SD, Standard Deviation

Figure S7: Cost of treatment: Forest plot comparison of outpatient and inpatient antimicrobial therapy by paediatric population


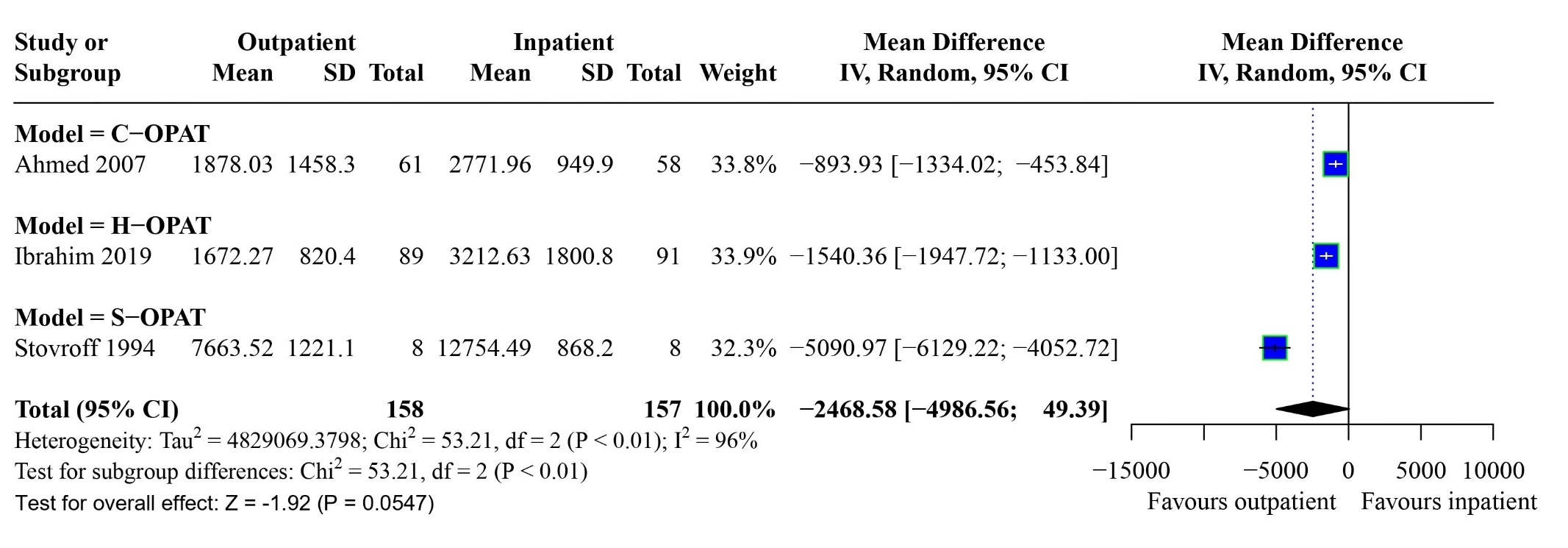


C-OPAT, outpatient clinic or infusion centre administration, S-OPAT, self or carer-administered OPAT at patients’ home; H-OPAT, physician or nurse-administered OPAT at patient home; IV, Inverse Variance; SD, Standard Deviation

Figure S8: Cost of treatment: Forest plot comparison of outpatient and inpatient antimicrobial therapy by discharge facilitation


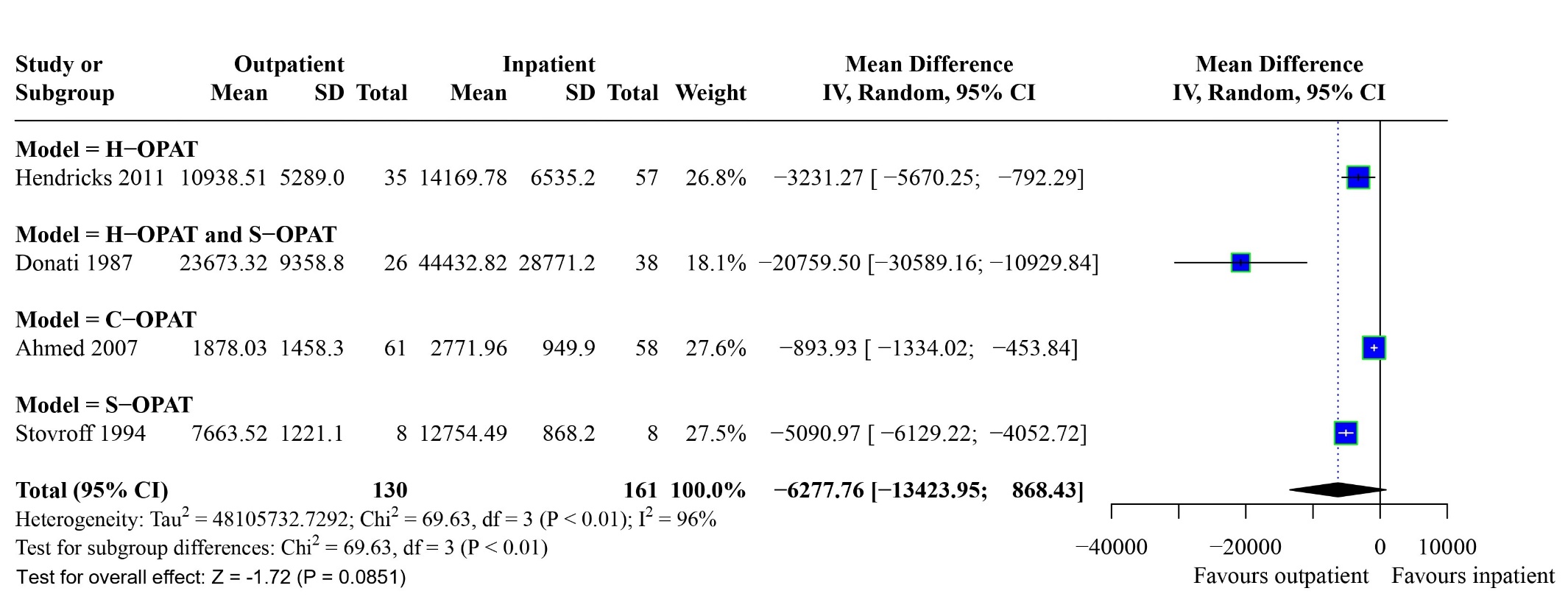
C-OPAT, outpatient clinic or infusion centre administration, S-OPAT, self or carer-administered OPAT at patients’ home; H-OPAT, physician or nurse-administered OPAT at patient home; IV, Inverse Variance; SD, Standard Deviation

Figure S9: Cost of treatment: Forest plot comparison of outpatient and inpatient antimicrobial therapy by admission avoidance


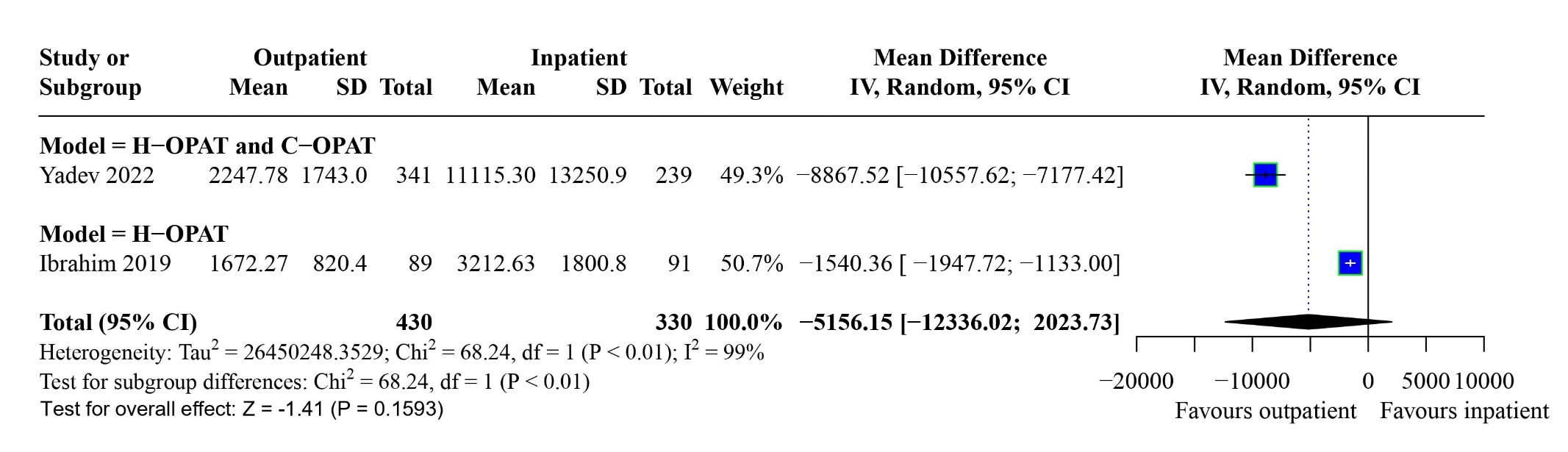
C-OPAT, outpatient clinic or infusion centre administration, S-OPAT, self or carer-administered OPAT at patients’ home; H-OPAT, physician or nurse-administered OPAT at patient home; IV, Inverse Variance; SD, Standard Deviation
